# Socioeconomic Disparities in Concussion Presentation

**DOI:** 10.1001/jamanetworkopen.2026.7416

**Published:** 2026-04-22

**Authors:** Daniel J. Corwin, Wenshan Li, Stephen G. Fung, Chengchun Yu, Chantal Backman, Monica Lamoureux, Steven Hawken, Sharon Johnston, Roger Zemek

**Affiliations:** 1 Division of Emergency Medicine, Children’s Hospital of Philadelphia, Philadelphia, Pennsylvania; 2 Perelman School of Medicine at the University of Pennsylvania, Philadelphia; 3 Ottawa Hospital Research Institute, Ottawa, Ontario, Canada; 4 Faculty of Health Sciences, University of Ottawa, Ontario, Canada; 5 Institute for Clinical Evaluative Sciences, Toronto, Ontario, Canada; 6 Institut du Savoir Montfort, Ottawa, Ontario, Canada; 7 Children’s Hospital of Eastern Ontario Research Institute, Ottawa, Ontario, Canada; 8 Department of Family Medicine, University of Ottawa, Ottawa, Ontario, Canada; 9 Department of Pediatrics and Emergency Medicine, University of Ottawa, Ottawa, Ontario, Canada

## Abstract

**Importance:**

The lack of a comprehensive population-level study evaluating the association of marginalization markers with concussion presentation limits opportunities for health care system improvements.

**Objective:**

To describe the association of socioeconomic measures of disparity with location of concussion presentation and follow-up rates.

**Design, Setting, and Participants:**

This population-based cohort study used linked administrative databases to assess patients with *International Statistical Classification of Diseases and Related Health Problems, Tenth Revision, Canada (ICD-10-CA) *concussion diagnosis codes at emergency department (ED) or outpatient clinics in Ontario, Canada, from April 1, 2010, to March 31, 2023. Data analysis was performed from March 1, 2025, to February 8, 2026.

**Exposures:**

Patient-level exposures were age, sex, immigrant status, presence of family physician, and rurality. Neighborhood-level exposures were income quintile, Ontario Marginalization (ON-Marg) Material Resource Index (access to basic material needs, such as percentage of unemployment), Household and Dwelling Index (accommodation type, such as percentage living alone), and Racialized and Newcomer Populations Index (such as percentage of recent immigrants or those who identify as a visible minority group) within 4 different age groups (<18, 18-39, 40-64, and ≥65 years).

**Main Outcomes and Measures:**

Location of initial visit (ED vs outpatient, including urgent care, walk-in, primary care, and specialty clinic) and presence of outpatient follow-up visit at 30 days or less.

**Results:**

Overall, 674 629 patients were evaluated (356 842 [52.9%] female; mean [SD] age, 32.8 [22.0] years). Marginalization measures were higher for patients first presenting to EDs vs outpatient via the ON-Marg Material Resources Index (57 043 [20.4%] vs 56 856 [14.4%]; difference, 6.2 [95% CI, 6.0-6.3] percentage points), rurality (47 356 [16.9%] vs 35 521 [9.0%]; difference, 7.9 [95% CI, 7.8-8.1] percentage points), and presence of a family physician (264 179 [94.4%] vs 388 346 [98.3%]; difference, −3.9 [95% CI, −3.8 to −4.0] percentage points). In modeling, having a family physician was associated with first seeking care in EDs (18-39 years: odds ratio [OR], 4.71; 95% CI, 4.41-5.03), as was living in rural areas (18-39 years: OR, 1.56; 95% CI, 1.51-1.62). Follow-up rates at 30 days or earlier were lower for patients first seen in EDs (24 307 [8.7%]) vs outpatient (110 821 [28.1%]). In regression analysis assessing variables associated with follow-up, first being seen in EDs (aged 65 years: OR, 0.21; 95% CI, 0.19-0.22), not possessing a family physician (aged ≥65 years: OR, 0.29; 95% CI, 0.19-0.46), and being in the most marginalized quintile for ON-Marg Material Resources (aged ≥65 years: OR, 0.74; 95% CI, 0.65-0.84) were all significantly associated with not completing a follow-up visit.

**Conclusions and Relevance:**

In this cohort study of patients with concussion treated in Ontario, Canada, those with higher marginalization markers were more likely to first seek care in EDs and have lower follow-up rates. These findings emphasize the importance of expanding health care system–wide resources, including primary care access and telemedicine, and enhancing resources for ED clinicians to optimize concussion care.

## Key Points

**Question:**

Within a province-wide Canadian dataset, what socioeconomic disparities are associated with the location of the first health care presentation for concussion?

**Findings:**

In this cohort study of 674 629 patients, increasing marginalization markers, specifically neighborhood socioeconomic measures, rurality, and absence of a primary care physician, were associated with increased likelihood of presenting to the emergency department rather than outpatient locations for concussion across multiple age ranges.

**Meaning:**

These findings emphasize the importance of augmenting health care system–wide resources, including primary care access, telemedicine, and streamlined education tools, in addition to enhancing resources for emergency department clinicians to optimize concussion care across all ages.

## Teaser

This cohort study of patients with concussion assesses whether individual socioeconomic and neighborhood-level markers of marginalization are associated with first location of concussion presentation and how follow-up visit rates differed based on initial health care system point of entry.

## Introduction

Concussion is a common injury across ages.^1,2^ Historically, concussion was managed with a one-size-fits-all approach of resting until symptom resolution.^3,4^ However, during the past decade, effective active therapeutic strategies^5,6^ initiated shortly after injury shifted the treatment paradigm to more individualized approaches.^7,8^ As personalized strategies evolve, treatment recommendations become more challenging for practitioners with limited time and resources to perform specialized evaluation technique, such as emergency department (ED) specialists.^9^

Amid concussion management challenges within EDs, prior work demonstrated substantial disparities in concussion presentation and follow-up across North America. In the US, prior work within individual health care systems found that patients with higher neighborhood-level markers of marginalization risk and racial and ethnic minority groups were more likely to be diagnosed with concussion in EDs compared with primary care locations.^10,11^ In several large Canadian provincial studies, disparities in patients’ ability to complete follow-up visits after concussion differed by socioeconomic and rurality statuses,^12^ and patients first evaluated by an ED clinician were less likely to complete follow-up care.^13^ However, these studies evaluated pediatric and adult patients separately, with many focusing on a single academic health care system. A comprehensive population-level study, at a provincial or statewide level, comparing individual- and neighborhood-level measures of marginalization for those patients first presenting to ED as opposed to an outpatient setting with concussion has yet to be performed. The lack of such a comprehensive study limits our ability to develop targeted yet highly impactful health care system–level changes at the regional and national levels to improve concussion care equity. Therefore, the goals of this study were to use population-level data to assess the association of individual socioeconomic and neighborhood-level markers of marginalization with first location of concussion presentation and to assess how follow-up visit rates differed based on initial health care system point of entry.

## Methods

We conducted a population-level, retrospective cohort study in Ontario, Canada. We used individually linked health administrative databases held at ICES (formally known as the Institute for Clinical Evaluative Sciences). This study was approved by the ICES Privacy and Compliance Office. ICES is a prescribed entity under section 45 of Ontario’s Personal Health Information Protection Act, which authorizes ICES to collect personal health information, without consent, for the purpose of analysis or compiling statistical information with respect to health care system management and planning.^14,15,16^ Data were collected and are reported in accordance with the Strengthening the Reporting of Observational Studies in Epidemiology (STROBE) reporting guideline.^17^

### Data Sources

The following datasets were linked and analyzed using unique encoded identifiers: the National Ambulatory Care Reporting System (NACRS), containing ED visit records; the Ontario Health Insurance Plan (OHIP) claims database, containing information on physician services provided; the Registered Persons Database, capturing sociodemographic information; the Ontario Census and the Postal Code Conversion File, containing neighborhood-level information and geographic identifiers; the Ontario Marginalization Index (ON-Marg) dataset, containing factor quintiles of a census-based index used to measure social marginalization^18^; and the Immigration, Refugees and Citizenship Canada (IRCC) permanent resident database to determine immigration status.

### Study Population

We included all individuals with *International Statistical Classification of Diseases and Related Health Problems, Tenth Revision, Canada (ICD-10-CA) *codes S060.x and OHIP diagnosis code 850 consistent with concussion diagnosis, regardless of concurrent injuries, and seen during an ED or outpatient visit from April 1, 2010, to March 31, 2023. For those with multiple concussions during the study period, the first concussion was used.^10,11^ Individuals were excluded if they had a previous visit for concussion 5 years or less from the index visit date, if they were ineligible for OHIP or were older than 105 years at the index date, or if they had missing or invalid sex or birth or death date (reported death before index visit). We stratified the cohort by age at index visit, given differing health care system needs and drivers among these age groups and anticipating that visit location and follow-up rates may differ across age groups: pediatric (<18 years), young adult (18-39 years), middle-aged adult (40-64 years), and older adult (≥65 years).^10,19,20^

### Individual- and Community-Level Markers

We captured individual-level markers of marginalization, including age, biological sex (as recorded on health card registration), immigrant status (immigration after 1985 per IRCC),^21^ presence of a family physician, rurality (towns or municipalities with population <10 000), and, for older adults, place of residence (as most long-term care facility residents are aged ≥65 years).^22^ We identified neighborhood-level markers of marginalization based on postal code, given the importance of neighborhood-level supports in concussion identification and management. In particular, we included composite neighborhood measures, which can provide a more holistic view of resources available to a patient.^10,23^ These markers include neighborhood income quintiles and 3 ON-Marg indices: (1) the Material Resources Index, derived from measures of access to and attainment of basic material needs; (2) the Household and Dwellings Index, derived from measures of residential accommodations; and (3) the Racialized and Newcomer Populations Index, derived from percentages of recent immigrants and those who self-identify as being in a minority group. We did not include the ON-Marg Age and Labour Force, which describes the percentage of older adults and the ratio of older adults and children to those aged 15 to 64 years, given prior work^24,25,26^ showing issues with age-only classifications of dependency, inconsistencies in prior studies using this index, and its lack of applicability to all age groups evaluated in our study. Neighborhood markers were taken from census data, obtained every 5 years (2011, 2016, and 2021 during the study period); the markers from the census year closest to the index visit were used for the analysis.

Additionally, we used the Health System Performance Research Network macro^14,15,16^ to identify those with mood disorders, other mental health disorders, stroke, and dementia in the 5 years preceding the index visit. These conditions are associated with concussion outcomes and may influence the initial location of care or reflect a more complex concussion presentation.^27,28,29^ As we were unable to obtain injury mechanism and chose not to measure concurrent injuries occurring in this population, we assessed the rate of admission from EDs as a surrogate of injury severity.

### Outcomes

The primary outcome was the initial location for concussion care (ED vs outpatient). Patients were determined to have first been seen in EDs if they only had an admission record in NACRS on the index visit date; patients were determined to have been seen in an outpatient clinic if they only had records in OHIP with location codes indicating outpatient settings, which include urgent cares, walk-in clinics, primary care clinics, and specialty clinics. If a patient had records in both settings on the same day, we considered outpatient setting as their initial location, assuming most would have been referred to EDs by their outpatient clinician. Our secondary outcome assessed whether patients had a follow-up visit in any outpatient setting within 30 days after the index visit, chosen given the definitions of persisting concussion symptoms.^5,30^

### Statistical Analysis

Descriptive statistics were used to describe demographic variables. We compared individual- and community-level markers of marginalization, stratified by initial location of visit, using a *z* test. We performed 4 logistic regressions (1 for each age group) to identify factors associated with initial visit location. All markers of marginalization were included in all 4 regressions (including age, as a linear term), except place of residence (used only for those aged ≥65 years). Comorbidities included mood disorders and other mental health disorders (all ages), stroke (all except those aged <18 years), and dementia (those aged 40-64 and ≥65 years). We conducted a sensitivity analysis in which patients with both ED and outpatient visits on the same day were recoded as having their first visit to the ED. We then conducted 4 separate logistic regressions to identify factors associated with the presence of a follow-up visit, with the same variables included (plus initial visit location). We used complete cases only because missingness was low (1.0%) and tied to postal code, making imputation difficult. Collinearity checks were conducted via the variance inflation factor, with no collinearity issues found across models. A 2-sided *P* < .05 was considered statistically significant. All analyses were performed with SAS software, version 9.4 (SAS Institute Inc). Data analysis was performed from March 1, 2025, to February 8, 2026.

## Results

In total, 674 629 patients (356 842 [52.9%] female and 317 787 [47.1%] male; mean [SD] age, 32.8 [22.0] years) were included in the analysis (Figure and Table 1). Of all patients, 22 271 (3.3%) had both ED and outpatient visits on the same day and were classified as first seeking outpatient care. Overall, 394 884 patients (58.5%) first sought care in outpatient settings. Of ED patients, 3844 (1.4%) were admitted to the hospital. The incidence of concussion increased each year from 2010 to 2019 (eFigure 1 in Supplement 1).

**Figure.**
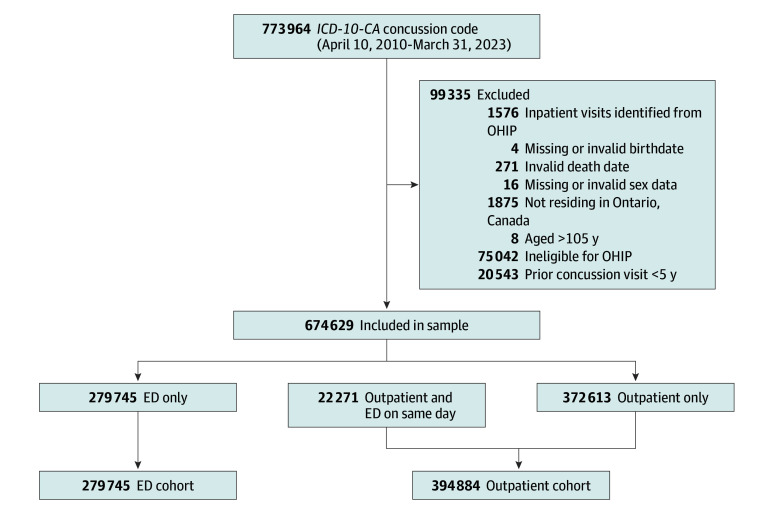
CONSORT Flow Diagram ED indicates emergency department; *ICD-10-CA*, *International Statistical Classification of Diseases and Related Health Problems, Tenth Revision, Canada*; OHIP, Ontario Health Insurance Plan.

**Table 1.** Demographic Characteristics of the Overall Cohort by Age Groups

Characteristic	No. (%) of patients by age group					*P* value
	Total (N = 674 629)	<18 y (n = 236 385)	18-39 y (n = 206 938)	40-64 y (n = 157 638)	≥65 y (n = 73 668)	
Sex						
Female	356 842 (52.9)	103 267 (43.7)	113 816 (55.0)	94 346 (59.8)	45 413 (61.6)	<.001
Male	317 787 (47.1)	133 118 (56.3)	93 122 (45.0)	63 292 (40.2)	28 255 (38.4)	
Presence of family physician						
Yes	652 525 (96.7)	225 682 (95.5)	200 406 (96.8)	154 094 (97.8)	72 343 (98.2)	<.001
No	22 104 (3.3)	10 704 (4.5)	6532 (3.2)	3544 (2.2)	1325 (1.8)	
Residence						
Community	653 941 (96.9)	232 526 (98.4)	206 270 (99.7)	155 234 (98.5)	59 911 (81.3)	<.001
CCC	238 (0.0)	0 (0.0)	16 (0.0)	54 (0.0)	168 (0.2)	
HDC	17 983 (2.7)	3859 (1.6)	643 (0.3)	2212 (1.4)	11 269 (15.3)	
LTC	2467 (0.4)	0 (0.0)	9 (0.0)	138 (0.1)	2320 (3.1)	
Mood disorder						
Active^a^	127 727 (18.9)	16 280 (6.9)	50 436 (24.4)	44 897 (28.5)	16 114 (21.9)	<.001
Inactive^a^	142 764 (21.2)	8041 (3.4)	43 109 (20.8)	60 848 (38.6)	30 766 (41.8)	
Not prevalent^a^	404 138 (59.9)	212 064 (89.7)	113 393 (54.8)	51 893 (32.9)	26 788 (36.4)	
Other mental health						
Active^a^	61 639 (9.1)	21 269 (9.0)	17 777 (8.6)	16 512 (10.5)	6081 (8.3)	<.001
Inactive^a^	112 806 (16.7)	23 803 (10.1)	37 769 (18.3)	34 539 (21.9)	16 695 (22.7)	
Not prevalent^a^	500 184 (74.1)	191 313 (80.9)	151 392 (73.2)	106 587 (67.6)	50 892 (69.1)	
Stroke						
Active^a^	3330 (0.5)	32 (0.0)	176 (0.1)	912 (0.6)	2210 (3.0)	<.001
Inactive^a^	6501 (1.0)	138 (0.1)	296 (0.1)	1608 (1.0)	4459 (6.1)	
Not prevalent^a^	664 798 (98.5)	236 215 (99.9)	206 466 (99.8)	155 118 (98.4)	66 999 (90.9)	
Dementia						
Active^a^	4437 (0.7)	0 (0.0)	0 (0.0)	79 (0.1)	4358 (5.9)	<.001
Inactive^a^	5723 (0.8)	0 (0.0)	0 (0.0)	642 (0.4)	5081 (6.9)	
Not prevalent^a^	664 469 (98.5)	236 385 (100.0)	206 938 (100.0)	156 917 (99.5)	64 229 (87.2)	

Abbreviations: CCC, continuing care community; HDC, hospital discounted care; LTC, long-term care.

^a^ Active indicates active history of condition in past 2 years; inactive, not active in past 2 years but a history of the condition; and not prevalent, no history of condition.

Comparing marginalization markers by initial visit location (Table 2), patients first presenting to EDs vs outpatient settings had larger percentages of highest marginalization by the ON-Marg Material Resources Index (57 043 [20.4%] vs 56 856 [14.4%]; difference, 6.2 [95% CI , 6.0-6.2] percentage points), and Households and Dwellings Index (57 938 [20.7%] vs 72 210 [18.3%]; difference, 2.6 [95% CI, 2.4-2.8] percentage points). Conversely, patients first presenting to EDs had smaller percentages of highest marginalization for ON-Marg Racialized and Newcomer Populations Index (53 049 [19.0%] vs 83 351 [21.1%]; difference, −2.1 [95% CI, −1.9 to −2.3] percentage points). Those first presenting to EDs vs outpatient were more likely to reside in rural areas (47 356 [16.9%] vs 35 521 [9.0%]; difference, 7.9 [95% CI, 7.8-8.1] percentage points) and were less like to have a family physician (388 346 [98.3%] vs 264 179 [94.4%]; difference, −3.9 [95% CI, −3.8 to −4.0] percentage points).

**Table 2.** Comparison of Marginalization Markers by Initial Visit Location

Marginalization marker	No. (%) of patients			Difference (95% CI), percentage points^a^	*P* value
	Total (N = 674 629)	First visit ED (n = 279 745)	First visit outpatient (n = 394 884)		
**Community markers of marginalization**					
ON-Marg Material Resources Index, %					
≤20 (Least marginalized)	162 712 (24.1)	55 804 (19.9)	106 908 (27.1)	−7.1 (−6.9 to −7.3)	<.001
21-40	150 536 (22.3)	57 620 (20.6)	92 916 (23.5)	−2.9 (−2.6 to −3.1)	
40-60	128 643 (19.1)	54 490 (19.5)	74 153 (18.8)	0.8 (0.6 to 1.0)	
61-80	112 225 (16.6)	51 200 (18.3)	61 025 (15.5)	3.0 (2.8 to 3.2)	
>80 (Most marginalized)	113 899 (16.9)	57 043 (20.4)	56 856 (14.4)	6.2 (6.0 to 6.3)	
Missing	6614 (1.0)	3588 (1.3)	3026 (0.8)	NA	
ON-Marg Racialized and Newcomer Populations Index, %					
≤20 (Least marginalized)	118 766 (17.6)	61 367 (21.9)	57 399 (14.5)	7.6 (7.4 to 7.8)	<.001
21-40	130 444 (19.3)	57 757 (20.6)	72 687 (18.4)	2.4 (2.2 to 2.6)	
41-60	137 936 (20.4)	52 603 (18.8)	85 333 (21.6)	−2.7 (−2.5 to −2.9)	
61-80	144 469 (21.4)	51 381 (18.4)	93 088 (23.6)	−5.2 (−5.0 to −5.4)	
>80 (Most marginalized)	136 400 (20.2)	53 049 (19.0)	83 351 (21.1)	−2.1 (−1.9 to −2.3)	
Missing	6614 (1.0)	3588 (1.3)	3026 (0.8)	NA	
ON-Marg Households and Dwellings Index, %					
≤20 (Least marginalized)	150 286 (22.3)	52 595 (18.8)	97 691 (24.7)	−5.9 (−5.7 to −6.1)	<.001
21-40	136 485 (20.2)	54 470 (19.5)	82 015 (20.8)	−1.2 (−1.0 to −1.4)	
41-60	127 352 (18.9)	54 829 (19.6)	72 523 (18.4)	1.4 (1.2 to 1.5)	
61-80	123 753 (18.3)	56 325 (20.1)	67 428 (17.1)	3.2 (3.0 to 3.4)	
>80 (Most marginalized)	130 139 (19.3)	57 938 (20.7)	72 201 (18.3)	2.6 (2.4 to 2.8)	
Missing	6614 (1.0)	3588 (1.3)	3026 (0.8)	NA	
Neighborhood income quintile					
Fifth (highest income)	161 413 (23.9)	55 717 (19.9)	105 696 (26.8)	−6.9 (−6.8 to −7.1)	<.001
Fourth	145 047 (21.5)	56 276 (20.1)	88 771 (22.5)	−2.4 (−2.2 to −2.6)	
Third	130 695 (19.4)	55 016 (19.7)	75 679 (19.2)	0.5 (0.3 to 0.7)	
Second	120 887 (17.9)	54 539 (19.5)	66 348 (16.8)	2.7 (2.5 to 2.9)	
First (lowest income)	114 074 (16.9)	57 055 (20.4)	57 019 (14.4)	6.0 (5.8 to 6.2)	
Missing	2513 (0.4)	1142 (0.4)	1371 (0.3)	NA	
**Individual markers of marginalization**					
Presence of family physician					
No	22 104 (3.3)	15 566 (5.6)	6538, (1.7)	NA	<.001
Yes	652 525 (96.7)	264 179 (94.4)	388 346 (98.3)	−3.9 (−3.8 to −4.0)	
Rurality of residence					
Urban	589 953 (87.4)	231 642 (82.8)	358 311 (90.7)	NA	<.001
Rural	82 877 (12.3)	47 356 (16.9)	35 521 (9.0)	7.9 (7.8 to 8.1)	
Missing	1799 (0.3)	747 (0.3)	1052 (0.3)	NA	
Immigrant status					
No	640 590 (95.0)	265 815 (95.0)	374 775 (94.9)	NA	.04
Yes	34 039 (5.0)	13 930 (5.0)	20 109 (5.1)	−0.1 (−0.0 to −0.2)	

Abbreviations: ED, emergency department; NA, not applicable; ON-Marg, Ontario Marginalization Index.

^a^ Differences and corresponding 95% CIs calculated from complete case analyses only.

In regressions for initial visit location (Table 3; eFigure 2 in Supplement 1), being most marginalized by income and the ON-Marg Material Resources Index was associated with first seeking care in EDs, with a dose-response association in each age group. For example, in those younger than 18 years, the odds ratio (OR) for the most vs least marginalized quintile seeking care in the ED was 1.58 (95% CI, 1.52-1.65) compared with 1.28 (95% CI, 1.23-1.32), 1.17 (95% CI, 1.13-1.20), and 1.04 (95% CI, 1.02-1.07) for the second, third, and fourth most marginalized quintiles, respectively. For the ON-Marg Racialized and Newcomer Population Index, being most marginalized was significantly associated with first seeking care in an outpatient setting, with a dose-response association in each age group. For example, in those younger than 18 years, the OR for most marginalized quintile was 0.61 for ED vs outpatient (95% CI, 0.59-0.63) vs 0.63 (95% CI, 0.61-0.65), 0.73 (95% CI, 0.71-0.75), and 0.87 (95% CI, 0.84-0.89) for the second, third, and fourth most marginalized quintiles, respectively. Across all age groups, having a family physician was associated with first seeking care in EDs (aged 18-39 years: OR, 4.71; 95% CI, 4.41-5.03) as was living in rural areas (aged 18-39 years: OR, 1.56; 95% CI, 1.51-1.62). Model estimates did not change substantially in the sensitivity analysis that recoded patients seen in both settings on the same day (eTable in Supplement 1).

**Table 3.** Logistic Regressions for the Outcome of First Visit for Concussion in the Emergency Department

Characteristic	OR (95% CI) by age group^a^			
	<18 y	18-39 y	40-64 y	≥65 y
Age	0.98 (0.98-0.98)	0.99 (0.99-0.99)	1.00 (1.00-1.00)	1.01 (1.01-1.01)
Sex				
Male	1.00 [Reference]	1.00 [Reference]	1.00 [Reference]	1.00 [Reference]
Female	0.93 (0.91-0.95)	0.84 (0.83-0.86)	0.89 (0.87-0.91)	0.96 (0.93-0.99)
Presence of family physician				
Yes	1.00 [Reference]	1.00 [Reference]	1.00 [Reference]	1.00 [Reference]
No	2.45 (2.35-2.56)	4.71 (4.41-5.02)	4.40 (4.05-4.77)	2.66 (2.35-3.02)
Residence				
Community	NA	NA	NA	1.00 [Reference]
CCC	NA	NA	NA	0.28 (0.19-0.41)
HDC	NA	NA	NA	1.30 (1.24-1.36)
LTC	NA	NA	NA	1.43 (1.30-1.57)
Rurality^b^				
Urban	1.00 [Reference]	1.00 [Reference]	1.00 [Reference]	1.00 [Reference]
Rural	1.92 (1.86-1.97)	1.56 (1.51-1.62)	1.45 (1.39-1.50)	1.35 (1.28-1.41)
Immigrant status				
No	1.00 [Reference]	1.00 [Reference]	1.00 [Reference]	1.00 [Reference]
Yes	1.11 (1.06-1.18)	1.10 (1.06-1.14)	1.19 (1.14-1.24)	1.00 (0.89-1.11)
ON-Marg Material Resources Index, %				
≤20 (Least marginalized)^b^	1.00 [Reference]	1.00 [Reference]	1.00 [Reference]	1.00 [Reference]
21-40	1.04 (1.02-1.07)	1.16 (1.12-1.19)	1.07 (1.03-1.11)	1.04 (0.99-1.09)
41-60	1.17 (1.13-1.20)	1.25 (1.21-1.19)	1.17 (1.13-1.21)	1.05 (0.99-1.10)
61-80	1.28 (1.23-1.32)	1.39 (1.34-1.44)	1.27 (1.22-1.33)	1.12 (1.06-1.19)
>80 (Most marginalized)	1.58 (1.52-1.65)	1.68 (1.61-1.75)	1.48 (1.41-1.55)	1.27 (1.18-1.36)
ON-Marg Racialized and Newcomer Populations Index, %				
≤20 (least marginalized)^b^	1.00 [Reference]	1.00 [Reference]	1.00 [Reference]	1.00 [Reference]
21-40	0.87 (0.84-0.89)	0.90 (0.88-0.93)	0.92 (0.88-0.95)	0.91 (0.87-0.96)
41-60	0.73 (0.71-0.75)	0.76 (0.73-0.78)	0.79 (0.76-0.82)	0.84 (0.80-0.88)
61-80	0.63 (0.61-0.65)	0.65 (0.63-0.68)	0.74 (0.71-0.77)	0.76 (0.74-0.82)
>80 (Most marginalized)	0.61 (0.59-0.63)	0.66 (0.65-0.69)	0.79 (0.76-0.83)	0.79 (0.75-0.83)
ON-Marg Households and Dwellings Index, %				
≤20 (Least marginalized)^b^	1.00 [Reference]	1.00 [Reference]	1.00 [Reference]	1.00 [Reference]
21-40	1.07 (1.04-1.10)	1.02 (0.99-1.05)	1.02 (0.99-1.06)	1.05 (0.99-1.10)
41-60	1.07 (1.04-1.10)	1.01 (0.98-1.04)	1.02 (0.99-1.06)	1.05 (1.00-1.11)
61-80	1.05 (1.02-1.09)	1.01 (0.98-1.05)	0.99 (0.96-1.03)	1.02 (0.96-1.08)
>80 (Most marginalized)	1.01 (0.97-1.05)	0.95 (0.92-0.98)	1.03 (0.99-1.07)	0.97 (0.91-1.02)
Income				
5 (Highest)^b^	1.00 [Reference]	1.00 [Reference]	1.00 [Reference]	1.00 [Reference]
4	1.16 (1.13-1.19)	1.12 (109-1.16)	1.09 (1.05-1.12)	1.09 (1.04-1.15)
3	1.27 (1.23-1.31)	1.16 (1.12-1.20)	1.10 (1.05-1.14)	1.10 (1.04-1.16)
2	1.29 (1.24-1.34)	1.18 (1.13-1.22)	1.13 (1.08-1.19)	1.11 (1.05-1.19)
1 (Lowest)	1.44 (1.38-1.52)	1.31 (1.25-1.38)	1.22 (1.15-1.29)	1.19 (1.10-1.29)
Mood disorders				
Not prevalent	1.00 [Reference]	1.00 [Reference]	1.00 [Reference]	1.00 [Reference]
Active	1.15 (1.11-1.19)	1.02 (1.00-1.05)	0.91 (0.88-0.94)	0.96 (0.92-1.00)
Inactive	1.05 (1.01-1.11)	1.09 (1.06-1.12)	0.96 (0.94-0.99)	0.96 (0.93-1.00)
Other mental health				
Not prevalent	1.00 [Reference]	1.00 [Reference]	1.00 [Reference]	1.00 [Reference]
Active	1.16 (1.12-1.19)	1.20 (1.16-1.24)	1.12 (1.08-1.16)	0.97 (0.92-1.03)
Inactive	1.05 (1.02-1.08)	1.16 (1.13-1.19)	1.09 (1.06-1.12)	1.03 (0.99-1.07)
Stroke				
Not prevalent	NA	1.00 [Reference]	1.00 [Reference]	1.00 [Reference]
Active	NA	0.64 (0.46-0.88)	0.79 (0.69-0.91)	0.92 (0.84-1.00)
Inactive	NA	1.08 (0.86-1.37)	1.33 (1.20-1.47)	1.13 (1.06-1.20)
Baseline dementia				
No	NA	NA	1.00 [Reference]	1.00 [Reference]
Yes	NA	NA	0.93 (0.81-1.07)	1.16 (1.10-1.21)

Abbreviations: CCC, continuing care community; HDC, hospital discounted care; LTC, long-term care; NA, not applicable; ON-Marg, Ontario Marginalization Index; OR, odds ratio.

^a^ Outcome indicates first visit for concussion in the emergency department (ie, the event); reference, first visit for concussion in outpatient setting (ie, nonevent).

^b^ Missing data for rurality, income, and ON-Marg: younger than 18 years, 1914 (0.9%); aged 18 to 39 years, 2747 (1.3%); aged 40 to 64 years, 1616 (1.0%); and 65 years or older, 639 (0.9%).

Those first seen in outpatient settings were more likely to follow up within 30 days (110 821 [28.1%]) vs EDs (24 307 [8.7%]; difference, 19.4 [95% CI, 19.2-19.5] percentage points). In regression analysis assessing variables associated with follow-up (Table 4), first being seen in EDs (aged 65 years: OR, 0.21; 95% CI, 0.19-0.22), not having a family physician (aged ≥65 years: OR, 0.29; 95% CI, 0.19-0.46), and being in the most marginalized quintile for the ON-Marg Material Resources Index (aged ≥65 years: OR, 0.74; 95% CI, 0.65-0.84) were all significantly associated with not completing a follow-up visit.

**Table 4.** Logistic Regressions for the Outcome of Outpatient Follow-Up Visit Within 30 Days of Index Visit

Characteristic	OR (95% CI) by age group, y^a^			
	<18	18-39	40-64	≥65
Initial visit location				
Outpatient	1.00 [Reference]	1.00 [Reference]	1.00 [Reference]	1.00 [Reference]
Emergency department	0.36 (0.36-0.37)	0.18 (0.18-0.19)	0.21 (0.20-0.21)	0.21 (0.19-0.22)
Age	1.10 (1.10-1.10)	1.00 (1.00-1.00)	0.98 (0.98-0.98)	0.96 (0.96-0.97)
Sex				
Male	1.00 [Reference]	1.00 [Reference]	1.00 [Reference]	1.00 [Reference]
Female	0.99 (0.97-1.00)	1.29 (1.26-1.32)	1.37 (1.33-1.41)	1.27 (1.20-1.35)
Presence of family physician				
Yes	1.00 [Reference]	1.00 [Reference]	1.00 [Reference]	1.00 [Reference]
No	0.94 (0.90-0.99)	0.59 (0.53-0.65)	0.47 (0.41-0.55)	0.29 (0.19-0.46)
Residence				
Community	NA	NA	NA	1.00 [Reference]
CCC	NA	NA	NA	2.51 (1.65-3.83)
HDC	NA	NA	NA	0.53 (0.47-0.59)
LTC	NA	NA	NA	0.41 (0.29-0.58)
Rurality^b^				
Urban	1.00 [Reference]	1.00 [Reference]	1.00 [Reference]	1.00 [Reference]
Rural	0.96 (0.93-1.00)	1.05 (1.00-1.10)	0.98 (0.94-1.03)	1.05 (0.96-1.16)
Immigrant status				
No	1.00 [Reference]	1.00 [Reference]	1.00 [Reference]	1.00 [Reference]
Yes	0.84 (0.79-089)	0.83 (0.79-0.87)	0.84 (0.79-0.88)	0.84 (0.69-1.03)
ON-Marg Material Resources Index, %				
≤20 (Least marginalized)^b^	1.00 [Reference]	1.00 [Reference]	1.00 [Reference]	1.00 [Reference]
21-40	0.97 (0.94-0.99)	0.93 (0.90-0.96)	0.97 (0.93-1.01)	0.92 (0.84-1.00)
41-60	0.90 (0.87-0.93)	0.88 (0.84-0.91)	0.89 (0.85-0.93)	0.84 (0.77-0.93)
61-80	0.84 (0.81-0.88)	0.81 (0.77-0.85)	0.83 (0.79-0.88)	0.82 (0.74-0.91)
>80 (Most marginalized)	0.76 (0.72-0.80)	0.74 (0.70-0.78)	0.74 (0.69-0.79)	0.74 (0.65-0.84)
ON-Marg Racialized and Newcomer Populations Index, %				
≤20 (Least marginalized)^b^	1.00 [Reference]	1.00 [Reference]	1.00 [Reference]	1.00 [Reference]
21-40	1.03 (1.00-1.07)	1.00 (0.96-1.05)	1.02 (0.98-1.07)	1.08 (0.99-1.18)
41-60	1.05 (1.01-1.09)	1.01 (0.97-1.06)	1.03 (0.98-1.08)	1.03 (0.94-1.12)
61-80	1.04 (1.01-1.09)	1.02 (0.98-1.06)	1.02 (0.97-1.07)	1.02 (0.93-1.13)
>80 (Most marginalized)	0.87 (0.84-0.91)	0.95 (0.91-1.00)	0.90 (0.86-0.95)	0.99 (0.89-1.10)
ON-Marg Households and Dwellings Index, %				
≤20 (Least marginalized)^b^	1.00 [Reference]	1.00 [Reference]	1.00 [Reference]	1.00 [Reference]
21-40	1.02 (1.00-1.05)	1.03 (0.99-1.09)	1.05 (1.01-1.09)	1.00 (0.91-1.10)
41-60	1.02 (0.99-1.05)	1.04 (1.00-1.09)	1.06 (1.02-1.11)	1.01 (0.92-1.12)
61-80	1.03 (0.99-1.07)	1.11 (1.06-1.16)	1.09 (1.04-1.140	1.06 (0.96-1.18)
>80 (Most marginalized)	1.10 (1.06-1.15)	1.16 (1.11-1.21)	1.10 (1.04-1.150	1.10 (0.99-1.22)
Income				
5 (Highest)^b^	1.00 [Reference]	1.00 [Reference]	1.00 [Reference]	1.00 [Reference]
4	0.93 (0.90-0.95)	0.98 (0.95-1.02)	0.99 (0.95-1.03)	0.96 (0.88-1.05)
3	0.89 (0.86-0.92)	0.96 (0.92-1.00)	0.99 (0.94-1.03)	1.03 (0.93-1.13)
2	0.86 (0.83-0.90)	0.94 (0.90-0.99)	0.99 (0.94-1.05)	0.96 (0.86-1.08)
1 (Lowest)	0.82 (0.78-0.87)	0.91 (0.85-0.97)	0.93 (0.86-1.00)	0.92 (0.80-1.06)
Mood disorders				
Not prevalent	1.00 [Reference]	1.00 [Reference]	1.00 [Reference]	1.00 [Reference]
Active	0.95 (0.91-0.98)	1.06 (1.03-1.09)	1.06 (1.02-1.10)	1.14 (1.05-1.23)
Inactive	0.94 (0.89-0.99)	1.04 (1.01-1.07)	1.12 (1.08-1.16)	1.16 (1.09-1.24)
Other mental health				
Not prevalent	1.00 [Reference]	1.00 [Reference]	1.00 [Reference]	1.00 [Reference]
Active	0.91 (0.88-0.95)	0.79 (0.76-0.83)	0.82 (0.78-0.86)	1.03 (0.93-1.14)
Inactive	0.98 (0.95-1.01)	0.94 (0.91-0.97)	0.96 (0.93-0.99)	1.03 (0.97-1.10)
Stroke				
Not prevalent	NA	1.00 [Reference]	1.00 [Reference]	1.00 [Reference]
Active	NA	0.46 (0.28-0.75)	0.49 (0.39-0.62)	0.91 (0.76-1.10)
Inactive	MA	0.54 (0.37-0.79)	0.77 (0.66-0.89)	0.84 (0.73-0.97)
Baseline dementia				
No	NA	NA	1.00 [Reference]	1.00 [Reference]
Yes	NA	NA	0.62 (0.49-0.77)	0.65 (0.56-0.74)

Abbreviations: CCC, continuing care community; HDC, hospital discounted care; LTC, long-term care; NA, not applicable; ON-Marg, Ontario Marginalization Index; OR, odds ratio.

^a^ Outcome indicates first visit for concussion in the emergency department (ie, the event); reference, first visit for concussion in outpatient setting (ie, nonevent).

^b^ Missing data for rurality, income, and ON-Marg: younger than 18 years, 1914 (0.9%); aged 18 to 39 years, 2747 (1.3%); aged 40 to 64 years, 1616 (1.0%); and 65 years or older, 639 (0.9%).

## Discussion

In this study, we found significant associations in presenting location for concussion with multiple markers of marginalization on a provincial level across multiple age groups. Specifically, individuals first presenting to EDs were significantly more likely to reside in marginalized neighborhoods with lower income and material and household resources compared with those first presenting to outpatient settings. We also found those first presenting to EDs to be significantly less likely to have a subsequent follow-up visit than those diagnosed in outpatient settings.

The higher proportion of patients with concussion from neighborhoods with limited material resources presenting to EDs is consistent with previous studies.^10,11^ Across a single health care system in the US, researchers found that pediatric patients living in neighborhoods with lower income levels and lower Child Opportunity Index scores (a composite neighborhood-level measure similar to ON-Marg) were more likely to first seek care in EDs when compared with outpatient locations.^10^ Although this may be due to more severe concussion presentations in these populations necessitating ED care, the fact that these disparities existed in the pediatric and younger adult populations, who, with the exception of very young infants, are at lower risk for intracranial injuries after head trauma and present with less complex concussion presentations,^29,31^ likely signifies that there are other contributing factors. Given the fact that many concussions can safely be managed in the outpatient setting, these differences may be related to either differences in knowledge or access to outpatient practitioners. Extensive prior work has shown that those with lower educational attainment and those with lower socioeconomic status are more likely to seek ED care for nonurgent issues.^32^ In addition, prior work has shown disparities for patients with lower socioeconomic status in accessing primary care in both the US and Canada,^33,34^ which may ultimately drive patients to first present to EDs.

Our observation that patients in rural areas more likely received ED care aligns with prior work.^35^ Specific to concussion, prior studies have found those in rural areas were less likely to have outpatient follow-up.^12^ As with material resources, the explanation for these differences may include more severe injuries^36,37^; however, care access likely plays an important role. Prior work related to care-seeking behavior among adolescent patients with concussion residing in rural areas has found that both issues with care access as well as disparities in knowledge underlie challenges in navigating the health care system.^38^ These findings highlight a need to improve care access for patients with concussion in rural communities. Several recent studies have described telehealth strategies, which may help close the rural-urban equity gap,^39,40^ and further investigation into telemedicine to help improve rural concussion care access is warranted.

Interestingly, a lower proportion of individuals with the highest ON-Marg Racialized and Newcomer Population Index scores first presented to EDs across all age groups. This finding was unexpected based on prior research of a US health care system, which found that patients identifying as non-Hispanic Black and Hispanic, when compared with non-Hispanic White, were more likely to present to EDs.^10^ Such disparities have also been found in studies of more general disease processes.^41^ Although these studies^10,41^ only evaluated US patients, prior work^42^ has found racial inequities in ED care in Canada, Australia, and New Zealand. Our findings may be influenced by patterns specific to Toronto, which, as an urban center, contains a high proportion of the minority and newcomer population. It is important to also note that, compared to our work, these prior studies^10,41,42^ evaluate race at the individual level. Our findings may be due to neighborhood diversity increasing engagement with outpatient practitioners, newcomers living in more heavily concentrated population densities, and/or a sociocultural connection to a family physician.^43^ Unfortunately, the existing datasets do not have race and ethnicity data available at the individual level, preventing a direct comparison with prior work. Further exploration of the interplay of race, ethnicity, and care-seeking behaviors is necessary.

Lastly, in evaluating follow-up patterns, presence of a primary care physician was a major factor associated with location of initial and follow-up care, consistent with prior work^44,45^ across disease conditions. Our low overall follow-up rates are consistent with prior province-wide studies in Canada.^12,13^ Although most patients (80%) did not have a follow-up visit within 30 days, an important consideration for concussion management and recovery,^7^ the rate of follow-up was approximately 3 times higher for patients first assessed in outpatient settings, and those without a family physician were up to 70% less likely to follow up. This finding may be because those choosing to present to EDs have less overall outpatient access. Poor follow-up rates underscore the challenges for ED clinicians in managing concussion but present an opportunity for researchers and clinicians to develop streamlined tools to assist ED clinicians in concussion discharge management.^30,46,47^ Efforts to raise community awareness and understanding of concussion, particularly by addressing markers of marginalization, are also critically important to help augment ED care. Ultimately, improving primary care access should be a priority to optimizing overall concussion care.

### Limitations

There are several limitations to the current study. As a study reliant on administrative databases, there was a potential for misclassification. We were unable to capture injury mechanism and did not include additional measures of injury severity, such as concurrent injuries, in our analysis. However, as only 1.4% of ED patients were admitted to the hospital, the likelihood of substantial unmeasured confounding due to injury severity is low. Although other unmeasured confounders might have influenced our findings, as our objective was to undertake a high-level evaluation of disparities influencing location of presentation and follow-up visit rates, we believe our findings still provide valuable information. Immigration status relied on those who first emigrated to Canada through Ontario; thus, a patient arriving in a different province would be misclassified. In addition, the IRCC database only extends to 1985 and therefore may misclassify older adults who emigrated prior to this date. It also does not distinguish immigrants from refugees, who may have distinct health care–seeking behaviors.^48^ As our databases do not distinguish clinic type (walk-in vs primary care vs specialty outpatient care), we were unable to further classify outpatient visit type. Our follow-up visit designation was based on administrative physician claims data; therefore, follow-up visits with nonphysicians (eg, physiotherapists) were not captured. Given our large sample size, we had multiple measures with statistical significance; as many of our markers were evaluated across quintiles, relevant trends hold clinical and societal meaning. Our measures of marginalization primarily focused on neighborhood-level measures and do not account for individual variation. Due to phenomena such as rapid gentrification and given reliance on census data obtained every 5 years that may not fully capture up-to-date neighborhood changes, these measures may be inaccurate for individual patients.^49^ We did not have access to individual race and ethnicity measures, precluding us from assessing the specific association between these markers and care location as well as evaluating the interplay between individual race and ethnicity and community diversity in care-seeking. Future studies should explore this interaction as well as examine factors, such as injury mechanism and visit timing, that may further advance our knowledge of factors influencing health care system engagement for concussion patients. Additionally, while a study strength was our province-wide analysis, the findings may not be generalizable to other provinces or countries.

## Conclusions

This study found that patients first seeking concussion care in EDs compared with outpatient settings were more likely to have greater markers of socioeconomic marginalization and be significantly less likely to attend a follow-up visit. These findings emphasize the importance of augmenting health care system–wide resources, including enhancing primary care access, telemedicine, and streamlined education tools, in addition to enhancing resources for ED clinicians, to optimize concussion care across all ages.

## Notes

Supplement 1. **eTable.** Sensitivity analysis, recoding patients seen in emergency department and outpatient settings on the same day as first seen in outpatient (rather than first seen in the emergency department) **eFigure 1.** Concussion incidence by study year stratified by age **eFigure 2.** Forest plots for models assessing the association of various individual- and neighborhood-level markers of marginalization with presenting to the emergency department following concussion, compared to the outpatient setting, across four age groups

Supplement 2. **Group Information.** Transforming Research by Assessing Neuroinformatics Across the Spectrum of Concussion by Embedding Interdisciplinary Data-Collection to Enable Novel Treatments (TRANSCENDENT) Research Program

Supplement 3.
Data Sharing Statement
