# Supplementary material for: Socioeconomic Disparities in Concussion Presentation
Source: JAMA Netw Open. 2026 Apr 22;9(4):e267416. doi: 10.1001/jamanetworkopen.2026.7416 (PMC13103813; doi:10.1001/jamanetworkopen.2026.7416)
Supplement: Supplement 1. — eTable. Sensitivity analysis, recoding patients seen in emergency department and outpatient settings on the same day as first seen in outpatient (rather than first seen in the emergency department) eFigure 1. Concussion incidence by study year stratified by age eFigure 2. Forest plots for models assessing the association of various individual- and neighborhood-level markers of marginalization with presenting to the emergency department following concussion, compared to the outpatient setting, across four age groups [file jamanetwopen-e267416-s001.pdf]

Supplemental Online Content

Corwin DJ, Li W, Fung SG, et al. Socioeconomic disparities in concussion presentation. *JAMA Netw Open*. 2026;9(4):e267416. doi:10.1001/jamanetworkopen.2026.7416

eTable. Sensitivity analysis, recoding patients seen in emergency department and outpatient settings on the same day as first seen in outpatient (rather than first seen in the emergency department)

eFigure 1. Concussion incidence by study year stratified by age

eFigure 2. Forest plots for models assessing the association of various individual- and neighborhood-level markers of marginalization with presenting to the emergency department following concussion, compared to the outpatient setting, across four age groups

This supplemental material has been provided by the authors to give readers additional information about their work.

© 2026 Corwin et al. *JAMA Network Open.*

eTable. Sensitivity analysis, recoding patients seen in emergency department and outpatient settings on the same day as first seen in outpatient (rather than first seen in the emergency department)

**Characteristic**	**OR (95% CI) by age group, y[a]**			
	**<18**	**18-39**	**40-64**	**≥65**
Age	0.97 (0.97-0.97)	0.99 (0.99-0.99)	1.01 (1.00-1.01)	1.02 (1.02-1.03)
Sex				
Male	1.00 [Reference]	1.00 [Reference]	1.00 [Reference]	1.00 [Reference]
Female	0.91 (0.89-0.92)	0.79 (0.78-0.80)	0.83 (0.81-0.85)	0.89 (0.86-0.92)
Presence of family physician				
Yes	1.00 [Reference]	1.00 [Reference]	1.00 [Reference]	1.00 [Reference]
No	2.66 (2.55-278)	6.81 (6.31-7.35)	5.81 (5.30-6.37)	4.51 (3.84-5.29)
Residence				
Community	NA	NA	NA	1.00 [Reference]
CCC	NA	NA	NA	0.19 (0.13-0.27)
HDC	NA	NA	NA	1.47 (1.40-1.54)
LTC	NA	NA	NA	2.41 (2.15-2.70)
Rurality[b]				
Urban	1.00 [Reference]	1.00 [Reference]	1.00 [Reference]	1.00 [Reference]
Rural	2.03 (1.97-2.09)	1.65 (1.59-1.71)	1.49 (1.44-1.55)	1.31 (1.25-1.38)
Immigrant Status				
No	1.00 [Reference]	1.00 [Reference]	1.00 [Reference]	1.00 [Reference]
Yes	1.11 (1.06-1.18)	1.11 (1.07-1.15)	1.17 (1.13-1.22)	0.97 (0.87-1.08)
ON-Marg Material Resources Index				
(<=20% [least marginalized])[b]	1.00 [Reference]	1.00 [Reference]	1.00 [Reference]	1.00 [Reference]
21%-40%	1.04 (1.01-1.06)	1.16 (1.12-1.19)	1.05 (1.02-1.09)	1.02 (0.97-1.07)
41%-60%	1.17 (1.14-1.21)	1.26 (1.22-1.30)	1.16 (1.11-1.20)	1.03 (0.98-1.09)
61%-80%	1.32 (1.28-1.37)	1.41 (1.36-1.47)	1.27 (1.21-1.32)	1.10 (1.04-1.17)
>80% (most marginalized)	1.66 (1.59-1.73)	1.73 (1.66-1.81)	1.52 (1.44-1.59)	1.26 (1.18-1.35)
ON-Marg Racialized and Newcomer Populations Index				
(<=20% [least marginalized])[b]	1.00 [Reference]	1.00 [Reference]	1.00 [Reference]	1.00 [Reference]
21%-40%	0.84 (0.82-0.87)	0.88 (0.85-0.91)	0.89 (0.86-0.92)	0.90 (0.86-1.08)
41%-60%	0.71 (0.69-0.73)	0.74 (0.71-0.76)	0.76 (0.74-0.79)	0.83 (0.79-0.87)
61%-80%	0.61 (0.60-0.63)	0.63 (0.61-0.65)	0.72 (0.69-0.75)	0.79 (0.75-0.83)
>80% (most marginalized)	0.62 (0.60-0.64)	0.65 (0.62-0.67)	0.79 (0.76-0.82)	0.83 (0.78-0.87)
ON-Marg Households and Dwellings Index				

© 2026 Corwin et al. *JAMA Network Open.*

(<=20% [least marginalized])[b]	1.00 [Reference]	1.00 [Reference]	1.00 [Reference]	1.00 [Reference]
21%-40%	1.06 (1.03-1.09)	1.01 (0.98-1.04)	1.02 (0.98-1.05)	1.05 (1.00-1.11)
41%-60%	1.06 (1.03-1.09)	1.00 (0.97-1.03)	1.02 (0.99-1.06)	1.07 (1.01-1.13)
61%-80%	1.04 (1.01-1.07)	0.99 (0.96-1.03)	0.99 (0.96-1.06)	1.03 (0.97-1.09)
>80% (most marginalized)	0.99 (0.95-1.02)	0.93 (0.90-0.96)	1.02 (0.98-1.06)	0.98 (0.92-1.04)
Income				
5 (highest)[b]	1.00 [Reference]	1.00 [Reference]	1.00 [Reference]	1.00 [Reference]
4	1.15 (1.12-1.18)	1.13 (1.09-1.16)	1.09 (1.05-1.13)	1.09 (1.04-1.15)
3	1.26 (1.22-1.30)	1.17 (1.13-1.21)	1.10 (1.06-1.15)	1.12 (1.06-1.18)
2	1.28 (1.24-1.33)	1.19 (1.15-1.24)	1.13 (1.08-1.18)	1.14 (1.07-1.21)
1	1.43 (1.37-1.51)	1.34 (1.27-1.40)	1.21 (1.12-1.28)	1.21 (1.12-1.31)
Mood disorders				
Not prevalent	1.00 [Reference]	1.00 [Reference]	1.00 [Reference]	1.00 [Reference]
Active	1.16 (1.12-1.21)	1.01 (0.99-1.04)	0.88 (0.86-0.91)	0.92 (0.88-0.96)
Inactive	1.07 (1.02-1.12)	1.09 (1.06-1.11)	0.94 (0.91-0.96)	0.93 (0.90-0.96)
Other Mental Health				
Not prevalent	1.00 [Reference]	1.00 [Reference]	1.00 [Reference]	1.00 [Reference]
Active	1.16 (1.12-1.19)	1.25 (1.21-1.29)	1.14 (1.10-1.18)	0.95 (0.90-1.00)
Inactive	1.05 (1.02-1.08)	1.17 (1.14-1.20)	1.09 (1.06-1.12)	1.01 (0.97-1.05)
Stroke				
Not prevalent		1.00 [Reference]	1.00 [Reference]	1.00 [Reference]
Active	NA	0.66 (0.49-0.90)	0.86 (0.75-0.99)	0.88 (0.81-0.96)
Inactive	NA	1.06 (0.83-1.34)	1.38 (1.25-1.53)	1.19 (1.11-1.27)
Baseline Dementia				
No	NA	NA	1.00 [Reference]	1.00 [Reference]
Yes	NA	NA	1.05 (0.92-1.21)	1.26 (1.20-1.33)

Abbreviations: CCC, continuing care community; HDC, hospital discounted care; LTC, long-1 term care; NA, not applicable; ON-Marg, Ontario Marginalization Index; OR, odds ratio.

[a]Outcome indicates first visit for concussion in the emergency department (ie, the event); reference, first visit for concussion in outpatient setting (ie non-event).

[b]Missing data for rurality, income, or and ON-Marg younger than 18 years, 1914 (0.9%); aged 18 to 39 years, 2747 (1.3%); aged 40 to 64 years, 1616 (1.0%); and 65 years or older, 639 (0.9%).

© 2026 Corwin et al. *JAMA Network Open.*

eFigure 1. Concussion incidence by study year stratified by age

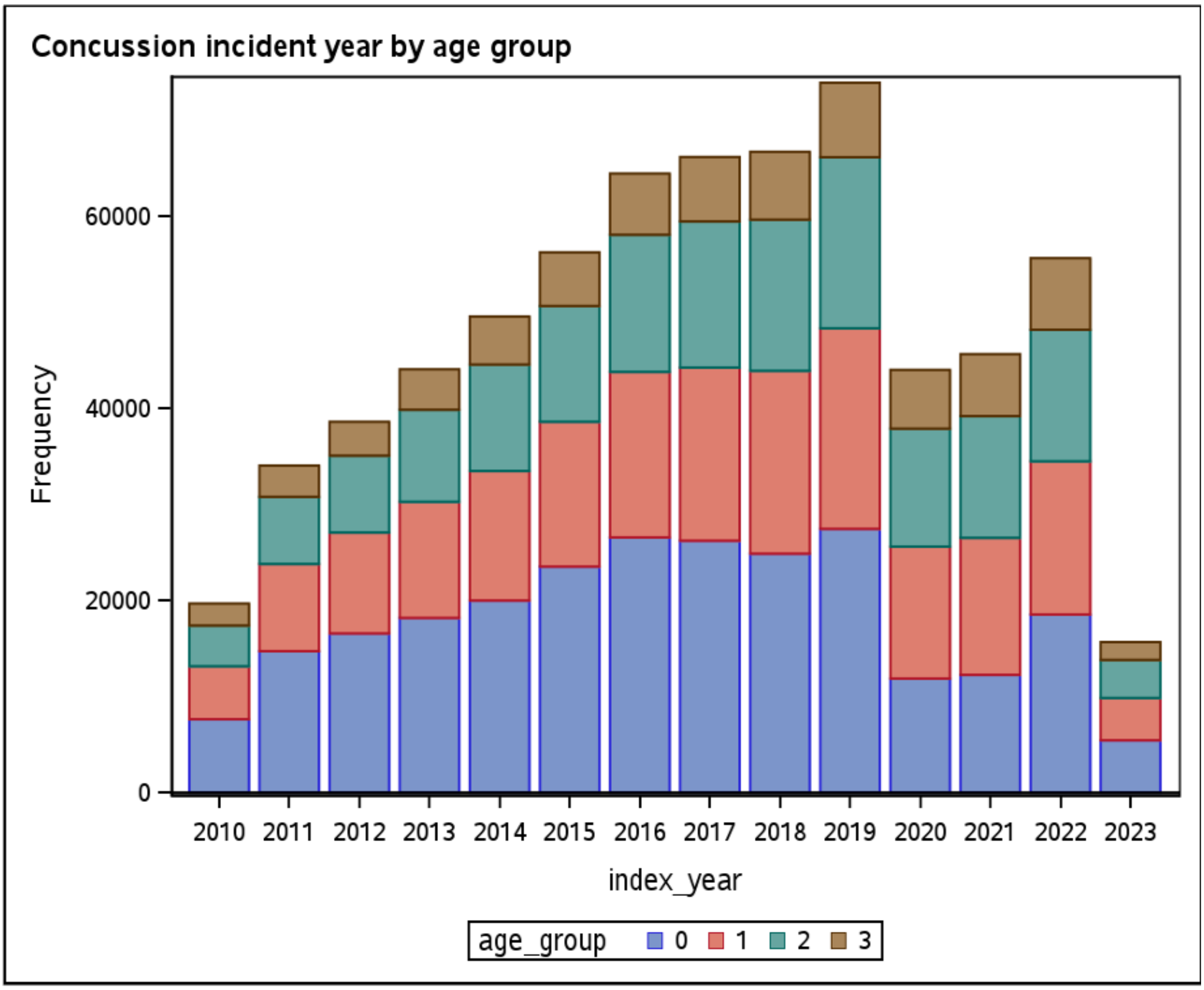

Incidence in concussion across the study population by study year, stratified by age
Age groups: 0 = <18 years; 1 = 18-39 years; 2 = 40-64 years; 3 = ≥65 years

© 2026 Corwin et al. *JAMA Network Open.*

eFigure 2a. Forest plot for model assessing the association of various individual- and neighborhood-level markers of marginalization with presenting to the emergency department following concussion, compared to the outpatient setting in patients <18 years

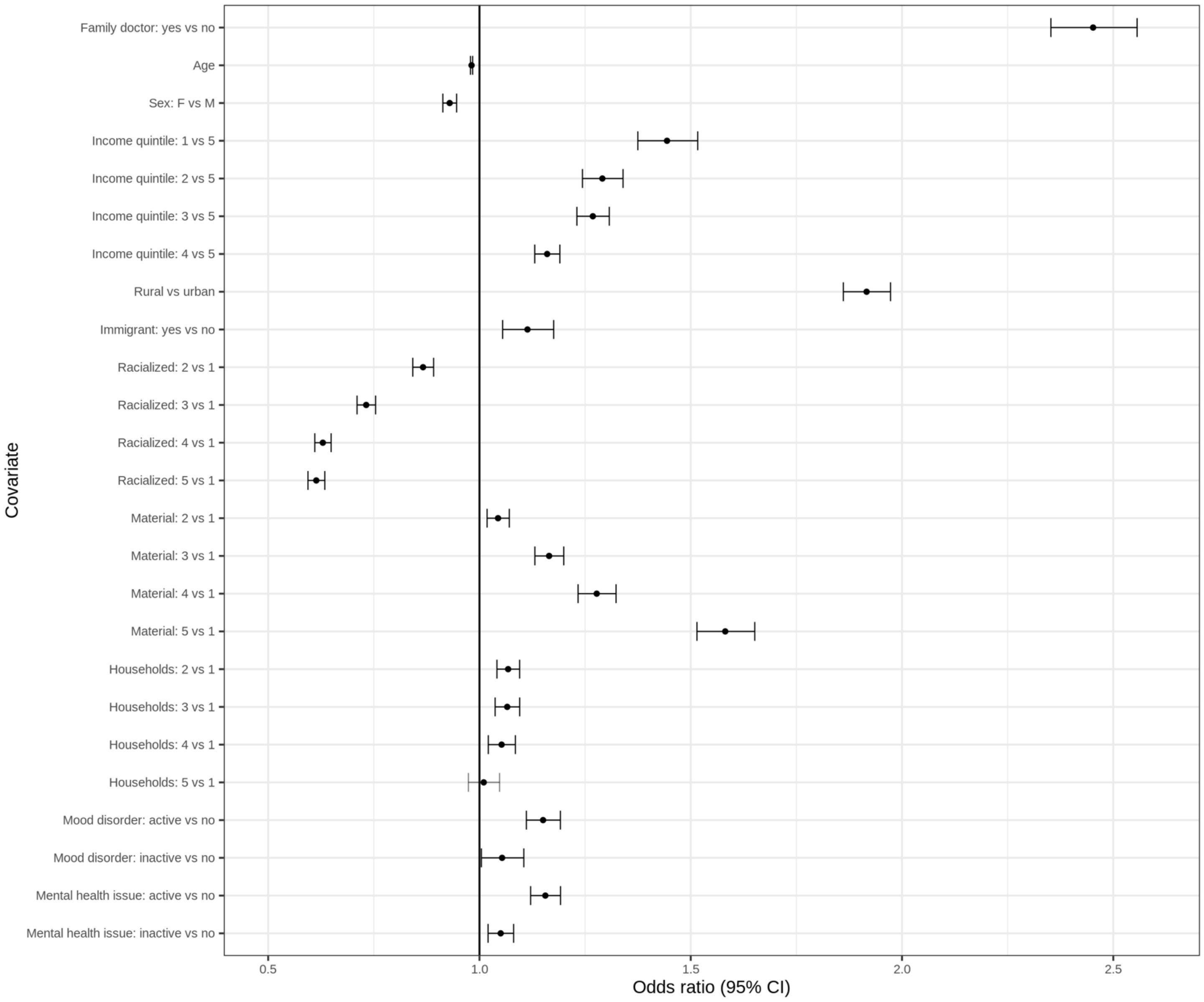

Income quintile: 5=highest neighborhood income quintile; Racialized: ON-Marg Racialized Populations index quintile, 1=least marginalized quintile; Material: ON-Marg Material Resources index quintile; Household: ON-Marg Household/Dwellings index quintile

© 2026 Corwin et al. *JAMA Network Open.*

eFigure 2b. Forest plot for model assessing the association of various individual- and neighborhood-level markers of marginalization with presenting to the emergency department following concussion, compared to the outpatient setting in patients 18-39 years

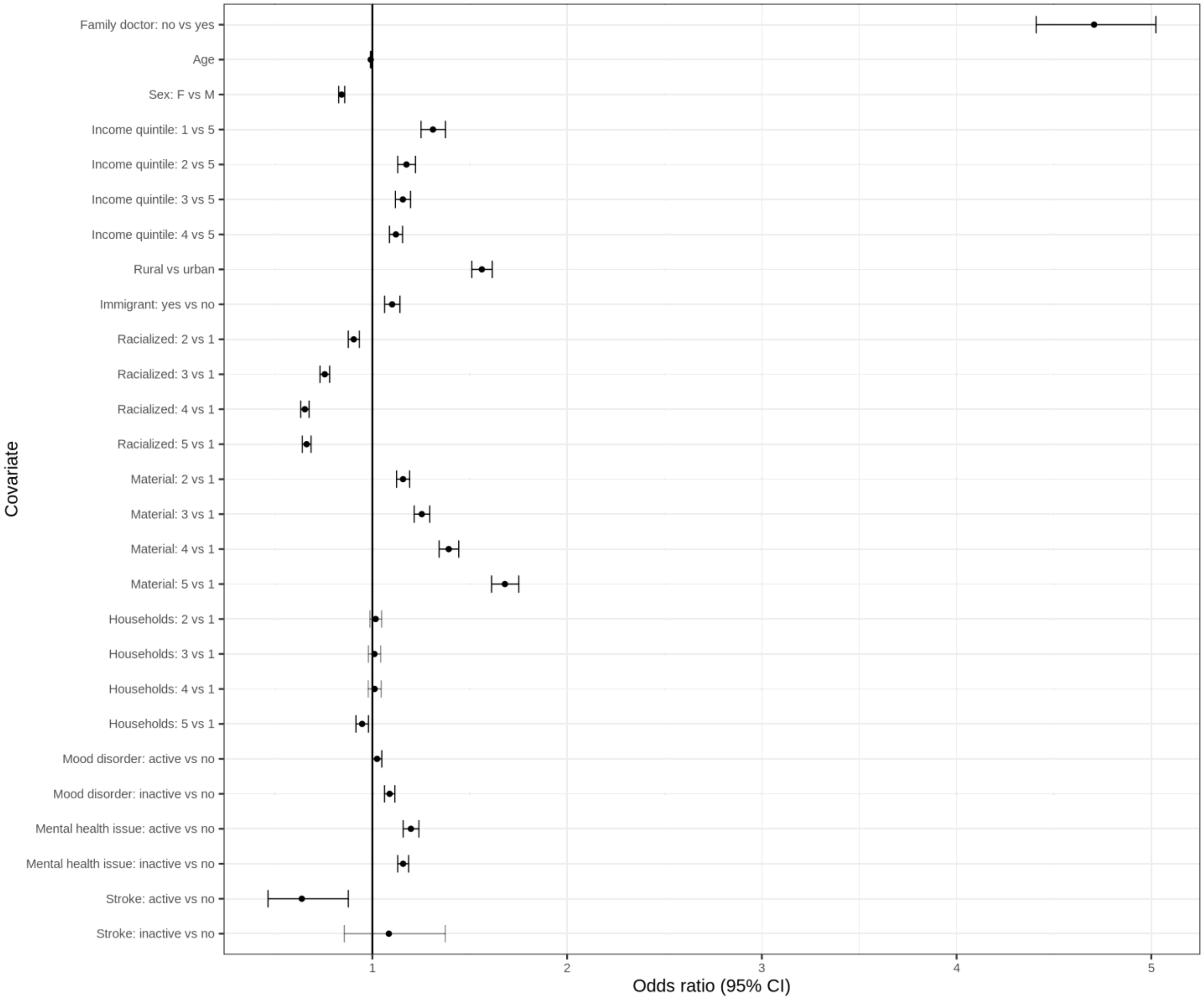

Income quintile: 5=highest neighborhood income quintile; Racialized: ON-Marg Racialized Populations index quintile, 1=least marginalized quintile; Material: ON-Marg Material Resources index quintile; Household: ON-Marg Household/Dwellings index quintile

© 2026 Corwin et al. *JAMA Network Open.*

eFigure 2c. Forest plot for model assessing the association of various individual- and neighborhood-level markers of marginalization with presenting to the emergency department following concussion, compared to the outpatient setting in patients 40-64 years

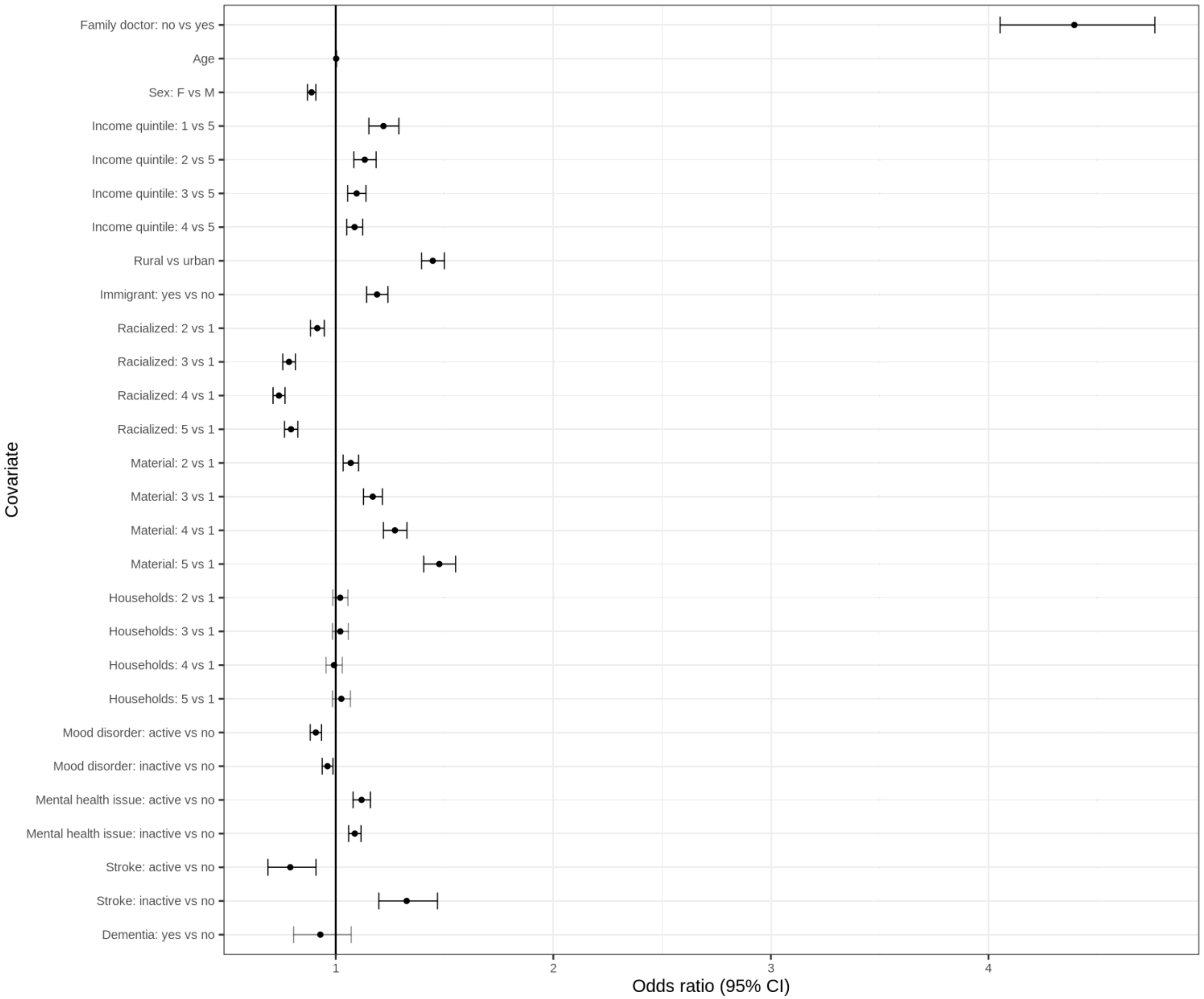

Income quintile: 5=highest neighborhood income quintile; Racialized: ON-Marg Racialized Populations index quintile, 1=least marginalized quintile; Material: ON-Marg Material Resources index quintile; Household: ON-Marg Household/Dwellings index quintile

© 2026 Corwin et al. *JAMA Network Open.*

eFigure 2d. Forest plot for model assessing the association of various individual- and neighborhood-level markers of marginalization with presenting to the emergency department following concussion, compared to the outpatient setting in patients ≥65 years

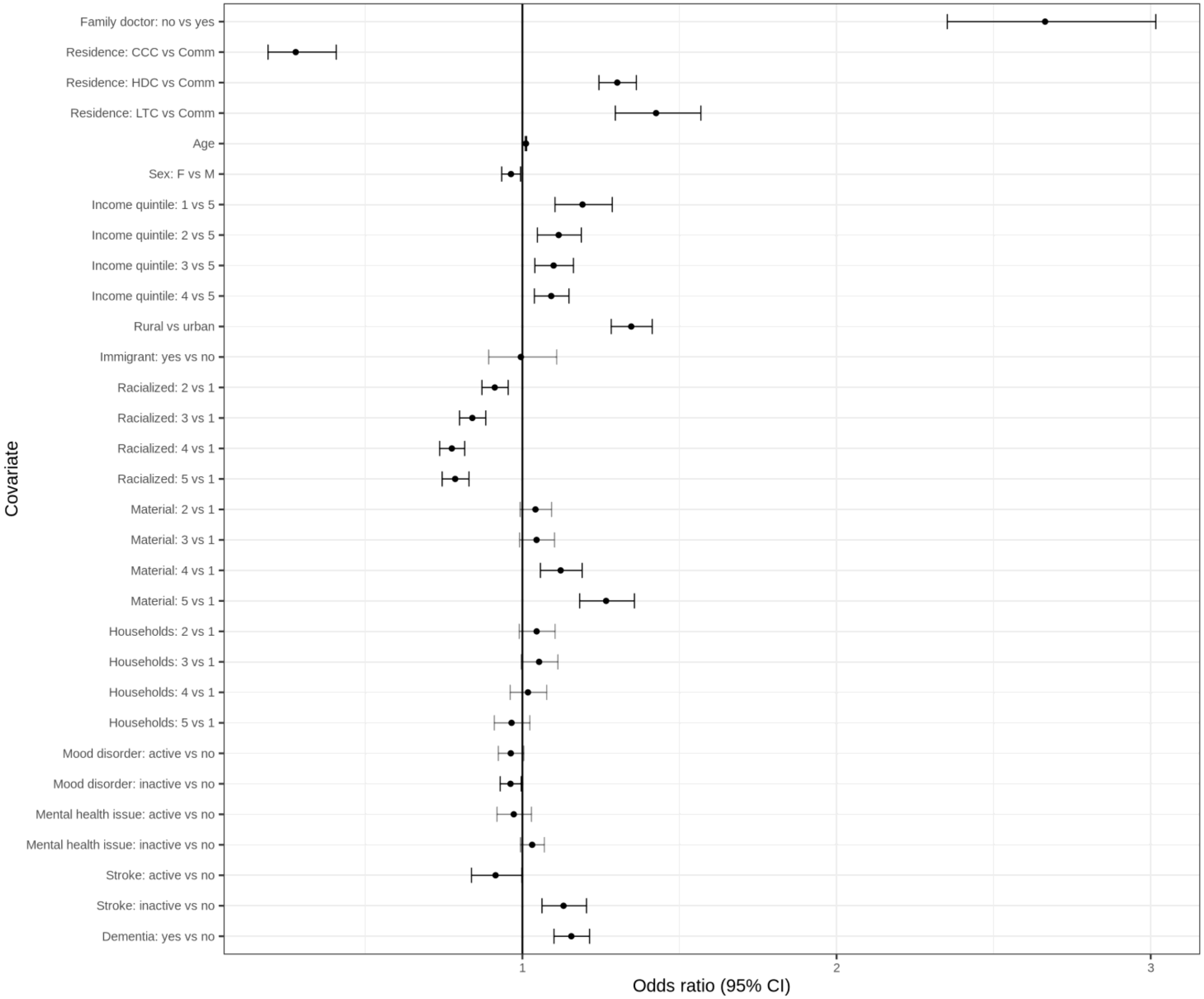

CCC: continuing care community; HDC: hospital discounted care; LTC: long-term care; Comm = community; Income quintile: 5=highest neighborhood income quintile; Racialized: ON-Marg Racialized Populations index quintile, 1=least marginalized quintile; Material: ON-Marg Material Resources index quintile; Household: ON-Marg Household/Dwellings index quintile

© 2026 Corwin et al. *JAMA Network Open.*
