# Supplementary material for: Socioeconomic Disparities in Concussion Presentation
Source: JAMA Netw Open. 2026 Apr 22;9(4):e267416. doi: 10.1001/jamanetworkopen.2026.7416 (PMC13103813; doi:10.1001/jamanetworkopen.2026.7416)
Supplement: Supplement 2. — Group Information. Transforming Research by Assessing Neuroinformatics Across the Spectrum of Concussion by Embedding Interdisciplinary Data-Collection to Enable Novel Treatments (TRANSCENDENT) Research Program [file jamanetwopen-e267416-s002.pdf]

Supplemental Online Content: Nonauthor Collaborators
*First name, last name, and suffix (if applicable) are required and will appear in PubMed.

*Group Name(s): Transforming Research by Assessing Neuroinformatics Across the Spectrum of Concussion by Embedding Interdisciplinary Data-Collection to Enable Novel Treatments (TRANSCENDENT) Research Program							
***First Name and Middle Initial(s)**	***Last Name**	***Suffix (eg, Jr, III)**	**Academic Degrees**	**Institution**	**Location (city, state/province, country)**	**Role or Contribution, eg, chair, principal investigator**	**Group (if more than 1 Group listed in the byline) and/or Subgroup (eg, Steering Committee)**
John J	Leddy		MD	Jacobs School of Medicine and Biomedical Sciences, University at Buffalo	Buffalo, New York, USA	co-PI	N/A
Andrée-Anne	Ledoux		PhD	Children’s Hospital of Eastern Ontario Research Institute / Faculty of Medicine, University of Ottawa	Ottawa, Ontario, Canada	co-PI	N/A
Nick	Reed		PhD, MScOT	Rehabilitation Sciences Institute, University of Toronto	Toronto, Ontario, Canada	co-PI	N/A
Noah D	Silverberg		PhD	Faculty of Medicine, University of British Columbia / Department of Psychology, University of British Columbia / Rehabilitation Research Program, Centre for Aging SMART, Vancouver Coastal Health Research Institute / Djavad Mowafaghian Centre for Brain Health	Vancouver, British Columbia, Canada	co-PI	N/A
Keith Owen	Yeates		PhD	Department of Psychology, Alberta Children's Hospital Research Institute / Hotchkiss Brain Institute, University of Calgary	Calgary, Alberta, Canada	co-PI	N/A
Peter	Tanuseputro		MD	University of Hong Kong Faculty of Medicine	Pokfulam, Hong Kong	co-I	N/A
