# Supplementary material for: Socioeconomic Disparities in Concussion Presentation
Source: JAMA Netw Open. 2026 Apr 22;9(4):e267416. doi: 10.1001/jamanetworkopen.2026.7416 (PMC13103813; doi:10.1001/jamanetworkopen.2026.7416)
Supplement: Supplement 3. — Data Sharing Statement [file jamanetwopen-e267416-s003.pdf]

Data Sharing Statement

Corwin. Socioeconomic Disparities in Concussion Presentation. *JAMA Netw Open*. Published April 22, 2026. doi:10.1001/jamanetworkopen.2026.7416

Data
Data available: Yes
Data types: Deidentified participant data, Data dictionary
How to access data: corwind@chop.edu
When available: With publication

Supporting Documents
Document types: Statistical/analytic code
How to access documents: corwind@chop.edu
When available: With publication

Additional Information
Who can access the data: Researchers whose proposed use of the data has been approved
Types of analyses: Approved secondary analyses
Mechanisms of data availability: After proposal approval and with a signed data access agreement
